# The effect of mirabegron on patient-related outcomes in patients with overactive bladder: the results of post hoc correlation and responder analyses using pooled data from three randomized Phase III trials

**DOI:** 10.1007/s11136-014-0904-4

**Published:** 2015-02-17

**Authors:** D. Castro-Diaz, C. R. Chapple, Z. Hakimi, M. B. Blauwet, L. Delgado-Herrera, W. Lau, S. Mujais

**Affiliations:** 1 Department of Urology, University Hospital of the Canary Islands, Ofra s/n. La Cuesta-38320-La Laguna, Santa Cruz de Tenerife, Spain; 2 Department of Urology, Royal Hallamshire Hospital, Sheffield, UK; 3 Astellas Pharma Global Development, Leiden, The Netherlands; 4 Astellas Pharma Global Development, Northbrook, IL USA

**Keywords:** Mirabegron, Overactive bladder, Patient-reported outcomes, Quality of life, Correlation

## Abstract

**Purpose:**

To understand how improvements in the symptoms of overactive bladder (OAB) seen with the β_3_-adrenoceptor agonist mirabegron 50 mg, correlate with patient experience as measured by validated and standard patient-reported outcomes (PROs), and to identify whether there is overall directional consistency in the responsiveness of PROs to treatment effect.

**Methods:**

In a post hoc analysis of pooled data from three randomized, double-blind, placebo-controlled, 12-week Phase III trials of mirabegron 50 mg once daily, responder rates for incontinence frequency (≥50 % reduction in incontinence episodes/24 h from baseline to final visit), micturition frequency (≤8 micturitions/24 h at final visit), and PROs [minimally important differences in patient perception of bladder condition (PPBC) and subsets of the overactive bladder questionnaire (OAB-q) measuring total health-related quality of life (HRQoL), and symptom bother] were evaluated individually and in combination.

**Results:**

Mirabegron 50 mg demonstrated greater improvement from baseline to final visit than placebo for each of the responder analyses, whether for individual objective and subjective outcomes or combinations thereof. These improvements versus placebo were statistically significant for all double and triple responder analyses and for all single responder analyses except PPBC. PRO measurements showed directional consistency and significant correlations, and there were also significant correlations between objective and subjective measures of efficacy.

**Conclusions:**

The improvements in objective measures seen with mirabegron 50 mg translate into a meaningful clinical benefit as evident by the directional consistency seen in HRQoL measures of benefit.

## Introduction

Overactive bladder (OAB) is defined by symptoms of urinary urgency, usually accompanied by frequency and nocturia, with or without urgency incontinence, in the absence of urinary tract infection (UTI) or other obvious pathology [1]. This condition is estimated to affect 12–17 % of adults in Europe and the USA [2–5]. OAB is associated with anxiety and depression [6–8], impairment of work productivity [9], and daily activities [10–12]. It is therefore associated with detrimental effects on health-related quality of life (HRQoL) [13]. Indeed, the severity of urgency urinary incontinence has been shown to be a predictor for HRQoL [14].

Clinical trials of treatments for the symptoms of OAB have traditionally relied on objective outcome measures to evaluate treatment efficacy, of which the most commonly used are those recorded in a patient bladder diary, namely micturition frequency, number of incontinence and urgency episodes in a 24 h period, number of nocturia episodes per 24 h, and volume voided per micturition [15, 16]. However, these endpoints say little about the impact that symptoms have on the patient’s quality of life (QoL) [17]. Indeed, it has been reported that improvements in objective outcomes do not necessarily correlate with improvements in subjective outcomes, that is, they do not necessarily translate into improvements in HRQoL [15, 16]. Consequently, there is a growing appreciation of the need to understand health outcomes from the patient’s point of view [18–21], particularly for the treatment of chronic debilitating conditions such as OAB, for which treatment is often aimed at symptom management rather than cure. The International Continence Society recommends that QoL measures be evaluated in the assessment of therapeutic interventions for the management of the symptoms of OAB [22].

A patient’s likelihood of persisting with a treatment for OAB is related to their satisfaction with that treatment [23]. Improvements in objective measures, such as micturition frequency, without a concomitant improvement in patient-reported measures of HRQoL, may not be sufficient to persuade the patient to persist with treatment. Indeed, improvements in objective outcomes achieved with common OAB drugs have typically not translated into long-term persistence; persistence rates ranging from 8 to 29 % have been reported in studies with at least 1 year of follow-up [24–27]. On the other hand, statistically significant improvements from baseline in the patient perception of bladder condition (PPBC) score have been seen in an open-label study of darifenacin along with treatment satisfaction for 85 % of patients [28].

Mirabegron is the first in a new class of agents—the β_3_-adrenoceptor agonists—to be approved for the treatment of OAB. In addition to objective bladder diary outcome measures, a range of PROs were evaluated in three randomized, double-blind, placebo-controlled, 12-week Phase III clinical trials (Studies 046 (NCT00689104) [29], 047 (NCT00662909) [30], and 074 (NCT00912964) [31]). Co-primary (objective) outcome measures in all three studies were change from baseline to final visit in mean number of micturitions per 24 h and mean number of incontinence episodes per 24 h. Patient-reported outcomes (PROs) included the overactive bladder questionnaire (OAB-q), the PPBC, and the treatment satisfaction visual analog scale (TS-VAS). In all three Phase III studies, and in a pre-specified, pooled analysis of the three studies [32], mirabegron 50 mg once daily resulted in statistically significant improvements from baseline to final visit versus placebo on both co-primary outcomes. It also resulted in a statistically significant improvement from baseline to final visit compared with placebo on PPBC as well as on the symptom bother scale and total HRQoL of the OAB-q in all three studies and the OAB-q subscales of coping and concern in Studies 046 and 047. Statistically significant improvement relative to placebo in the OAB-q subscale of sleep was also reported with mirabegron 50 mg in Study 047. In the pooled analysis, the only PRO reported was the TS-VAS; this too showed a statistically significant improvement from baseline to final visit for mirabegron 50 mg versus placebo. Analysis of the pooled data has also shown greater improvement in the EQ-5D utility score with mirabegron 50 mg compared with placebo and tolterodine [33].

This paper presents the results of post hoc analysis conducted on the large pooled mirabegron 50 mg and placebo data sets described above to determine correlations between various objective and subjective outcome measures utilized in the three Phase III trials. (The 25 mg dose was not evaluated in this post hoc analysis as it was used in Study 074 only and hence was not part of the pooled analysis.) Data showing the effects of mirabegron 50 mg on measures of HRQoL are also presented. In a novel approach to understanding these data, responder analyses were performed to assess the proportion of patients who were simultaneously responders for incontinence episodes and micturitions along with one or two different PROs at final visit. The aims were twofold: firstly, to understand how improvements in objective measures with mirabegron 50 mg correlate with the patient’s experience as measured by validated, standard PRO instruments and secondly, to understand how improvements in different PRO outcomes correlate with one another. This is of interest because PROs assess different components of the response of patients, resulting in heterogeneity in responsiveness to treatment effect.

## Methods

### Study design

An overview of the design of Studies 046, 047, and 074 is provided in Table 1. Importantly, all three studies were of identical design, the only difference being the inclusion of tolterodine as an active control in Study 046 (but not Studies 047 or 074) and the use of mirabegon 25 mg, but not mirabegron 100 mg in Study 074.

**Table 1:** Overview of design of Studies 046, 047, and 074

Study	Location	Study design	Duration of treatment^†^	Inclusion criteria^†^	Treatment groups
046	189 sites in Australia and Europe	046: Phase III, randomized, double-blind, placebo- and active-controlled study to evaluate the efficacy and safety of mirabegron	2-week single-blind, placebo run-in period followed by 12-week double-blind treatment period	Female and male adults aged ≥18 years who had symptoms of OAB (urinary frequency and urgency with or without incontinence) for ≥3 months and ≥8 micturitions per 24 h during 3-day micturition diary period collected during run-in period and ≥3 urgency episodes (PPIUS scale grade 3 or 4), with or without incontinence during 3-day micturition diary period collected during run-in period	Placebo Mirabegron 50 mg od Mirabegron 100 mg od Tolterodine ER 4 mg od
047	132 sites in Canada and USA	047 and 074: Phase III, randomized, double-blind, placebo-controlled studies to evaluate the efficacy and safety of mirabegron			Placebo Mirabegron 50 mg od Mirabegron 100 mg od
074	151 sites in Canada, USA, and Europe				Placebo Mirabegron 25 mg od Mirabegron 50 mg od

*OAB* overactive bladder, *od* once daily, *PPIUS* Patient Perception of Intensity of Urgency Scale

^†^All three studies

### Efficacy assessments

#### Objective measures

Co-primary efficacy outcome measures in all three studies were change from baseline to final visit in mean number of incontinence episodes per 24 h and change from baseline to final visit in mean number of micturitions per 24 h. A responder for incontinence episodes was defined as a patient who had incontinence at baseline and a ≥50 % decrease from baseline to final visit in mean number of incontinence episodes per 24 h. A responder for micturition frequency was defined as a patient with ≤8 micturitions per 24 h at final visit. (Note that the results of a responder analysis of incontinence frequency have been reported for Studies 046 and 074 [29, 31] as have the results of responder analyses of both incontinence and micturition frequency in a pooled analysis of all three studies [32].)

#### Subjective measures

Patient-reported outcomes evaluated in all three studies include change from baseline to final visit in PPBC, OAB-q symptom bother scale, OAB-q total HRQoL, and the OAB-q subscales of coping, concern, sleep, and social interaction. The PPBC was developed as a global assessment of bladder condition that asks patients to rate their subjective impression of their current bladder condition on a 6-point Likert scale, ranging from 1 (“my bladder condition does not cause me any problems at all”) to 6 (“my bladder condition causes me many severe problems”). The OAB-q symptom bother scale measures level of bother associated with OAB symptoms and is assessed using the eight items that comprise the symptom bother scale of the 33-item OAB-q. Total HRQoL, measured using the remaining 25 items of the OAB-q, is comprised of four subscales—coping, concern, sleep, and social interaction. Both the PPBC and the OAB-q have been validated in clinical and community settings and have demonstrated reliable internal consistency, test–retest reliability, construct validity, and responsiveness among patients with a range of OAB symptoms [34–38].

Patient perception of bladder condition was assessed at baseline and week 12/final visit. Higher scores on the PPBC indicate a poorer perception of bladder condition and negative values for change from baseline scores indicate improvement. OAB-q was assessed at baseline and weeks 4, 8, and 12/final visit, and scores transformed onto a 0–100 scale. OAB-q total HRQoL and subscale scores are directly related to patient wellbeing with higher scores indicating better QoL and a positive change in scores indicating improvement. The score on the OAB-q symptom bother scale is directly related to the degree of patient discomfort (bother) with the symptoms of OAB. Hence, lower scores on this scale indicate a better QoL, and a negative change in the symptom bother scale indicates improvement.

For the responder analysis of PROs, a responder was defined as a patient who achieved a change from baseline to final visit that exceeded the minimally important difference (MID), which is defined as “the smallest difference in score in the domain of interest that patients perceive as beneficial and which would mandate, in the absence of troublesome side effects and excessive costs, a change in patient management” [39]. The MID has been defined to be 10 points for OAB-q (symptom bother, total HRQoL, and subscales) based on anchor and distribution methods [34, 40, 41] and 1 point for PPBC [38].

### Statistical analyses

The full analysis set (FAS) in all three studies comprised all randomized patients who received at least one dose of study drug and had at least one micturition measurement in a 3-day micturition diary at baseline and at least one post-baseline diary. The FAS-incontinence (FAS-I) population comprised FAS patients who also recorded at least one incontinence episode in the 3-day baseline diary. Data for the placebo and mirabegron 50 mg arms of all three studies were pooled and change from baseline data analyzed using analysis of covariance (ANCOVA) with treatment group, sex, and study as fixed factors and baseline as a covariate. Pooling of the data was facilitated by the fact that all three studies were identical in design, with the same inclusion and exclusion criteria and outcome measurements (Table 1). Responder rates for PROs, individually and in combination with responder rates for incontinence frequency and micturition frequency, were determined: the double responder analysis involved evaluation of the proportions of patients who were simultaneously responders for either incontinence or micturitions as well as PPBC, OAB-q symptom bother scale, or OAB-q total HRQoL; the triple responder analysis involved evaluation of the proportions of patients who were simultaneously responders for either incontinence or micturitions as well as PPBC and OAB-q symptom bother scale or PPBC and OAB-q total HRQoL. Two-sided 95 % confidence intervals (CIs) for the differences in the proportions of responders between mirabegron 50 mg and placebo are based on normal approximation. Odds ratios, corresponding two-sided 95 % CIs, and *P* values were derived from a logistic regression model including treatment group, sex, study, and baseline value(s).

Spearman rank partial correlation coefficients, adjusted for baseline, between the objective endpoints (change from baseline to final visit in mean number of incontinence episodes per 24 h and mean number of micturitions per 24 h) and each of the subjective outcomes (OAB-q symptom bother scale, OAB-q total HRQoL, and PPBC) were derived. Additionally, Spearman rank partial correlation coefficients, adjusted for baseline, between change from baseline to final visit in PPBC and change from baseline to final visit in both the OAB-q symptom bother scale and total HRQoL were derived.

## Results

### Study population

The pooled placebo and mirabegron 50 mg groups consisted of 1,328 and 1,324 patients in the FAS, respectively, and 878 and 862 in the FAS-I, respectively. Patient demographics and baseline characteristics were similar in the pooled placebo and pooled mirabegron 50 mg treatment groups (Table 2).

**Table 2:** Patient demographics and baseline characteristics by pooled treatment group (FAS)

	Placebo (*n* = 1,328)	Mirabegron 50 mg (*n* = 1,324)
Females [*n* (%)]	966 (72.7)	942 (71.1)
Age (years), mean (SD)	59.2 (13.2)	59.7 (12.6)
Race [*n* (%)]		
White	1,227 (92.4)	1,235 (93.3)
Black or African-American	80 (6.0)	61 (4.6)
Asian	13 (1.0)	17 (1.3)
Other	8 (0.6)	11 (0.8)
Type of OAB [*n* (%)]		
Urgency incontinence only	442 (33.3)	491 (37.1)
Mixed stress/urgency incontinence	415 (31.3)	412 (31.1)
Frequency/urgency without incontinence	471 (35.5)	421 (31.8)
Duration of OAB (months), mean (SD)	86.3 (99.1)	85.2 (93.1)
Number of incontinence episodes per 24 h, mean (SD)	1.8 (2.5)	1.8 (2.5)
Number of micturitions per 24 h, mean (SD)	11.6 (3.1)	11.7 (3.2)

*FAS* full analysis set, *OAB* overactive bladder, *SD* standard deviation

### Efficacy

#### PRO outcomes

**Fig. 1:**
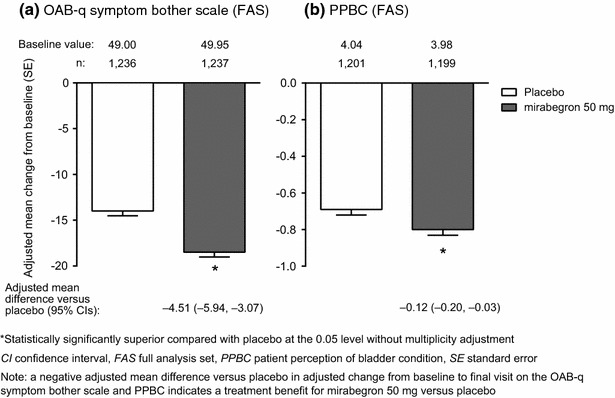
Adjusted mean change (SE) from baseline to final visit in **a** the OAB-q symptom bother scale (FAS) and **b** PPBC (FAS) with mirabegron 50 mg and placebo (pooled data)

**Fig. 2:**
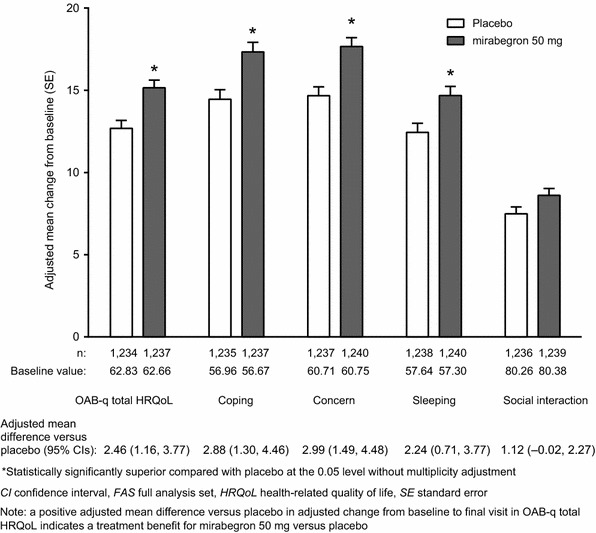
Adjusted mean change (SE) from baseline to final visit in OAB-q total HRQoL and its subscales of coping, concern, sleeping, and social interaction with mirabegron 50 mg and placebo (FAS; pooled data)

Mirabegron 50 mg resulted in a statistically significant improvement from baseline to final visit relative to placebo in the OAB-q symptom bother scale and PPBC (*P* < 0.05, Fig. 1). It also resulted in statistically significant improvements from baseline to final visit relative to placebo in OAB-q total HRQoL and the OAB-q subscales of coping, concern, and sleeping, but not social interaction (*P* < 0.05, Fig. 2).

#### Responder analyses

The percentage of subjects in the mirabegron 50 mg and placebo groups who were responders (i.e., achieved the MID) at final visit was: 66.0 and 57.8 %, respectively, for the OAB-q symptom bother scale, 56.8 and 48.9 % for OAB-q total HRQoL, and 55.9 and 53.1 % for PPBC. Thus, responder rates for the OAB-q symptom bother scale, OAB-q total HRQoL, and PPBC were numerically larger with mirabegron 50 mg than placebo; the odds ratios for mirabegron 50 mg versus placebo on responder rates were all greater than one (Table 3). The difference in responder rates between mirabegron 50 mg and placebo was statistically significant for the OAB-q symptom bother scale and OAB-q total HRQoL (*P* < 0.001), and it approached, but did not reach, statistical significance for PPBC. (Responder rates for incontinence and micturition frequency were also statistically significantly larger with mirabegron 50 mg than placebo; these results have been reported previously [32] and are shown in Table 3 for completeness.)

**Table 3:** Single, double, and triple responder analyses for the pooled mirabegron 50 mg group versus placebo

Responders^a^ for:	Difference versus placebo % (95 % CIs)^b^	Odds ratio (95 % CIs)^c^	*P* value^c^
**Single responder criterion**			
Incontinence (FAS-I)	9.9 (5.5, 14.4)	1.54 (1.26, 1.89)	<0.001
Micturitions (FAS)	7.0 (3.6, 10.4)	1.57 (1.30, 1.89)	<0.001
PPBC (FAS)	2.8 (−1.2, 6.7)	1.18 (0.99, 1.39)	0.059
OAB-q symptom bother score (FAS)	8.3 (4.5, 12.1)	1.43 (1.21, 1.70)	<0.001
OAB-q total HRQoL (FAS)	7.9 (4.0, 11.8)	1.46 (1.23, 1.74)	<0.001
**Double responder criteria**			
Incontinence and PPBC (FAS-I)	6.8 (1.9, 11.6)	1.37 (1.11, 1.68)	0.003
Incontinence and OAB-q symptom bother score (FAS-I)	15.2 (10.4, 20.0)	1.87 (1.53, 2.29)	<0.001
Incontinence and OAB-q total HRQoL (FAS-I)	10.9 (6.1, 15.7)	1.60 (1.30, 1.97)	<0.001
Micturitions and PPBC (FAS)	3.9 (0.8, 7.1)	1.42 (1.14, 1.77)	0.001
Micturitions and OAB-q symptom bother score (FAS)	6.7 (3.4, 10.0)	1.59 (1.30, 1.95)	<0.001
Micturitions and OAB-q total HRQoL (FAS)	6.0 (3.0, 9.1)	1.63 (1.31, 2.02)	<0.001
**Triple responder criteria**			
Incontinence and PPBC and OAB-q symptom bother score (FAS-I)	9.1 (4.2, 13.9)	1.55 (1.24, 1.93)	<0.001
Incontinence and PPBC and OAB-q total HRQoL (FAS-I)	8.4 (3.6, 13.2)	1.51 (1.20, 1.90)	<0.001
Micturitions and PPBC and OAB-q symptom bother score (FAS)	3.5 (0.4, 6.6)	1.42 (1.12, 1.79)	0.003
Micturitions and PPBC and OAB-q total HRQoL (FAS)	3.6 (0.7, 6.5)	1.47 (1.15, 1.88)	0.002

*CI* confidence interval, *FAS* full analysis set, *FAS*-*I* FAS-incontinence, *HRQoL* health-related quality of life, *PPBC* patient perception of bladder condition

^a^Responder definitions: incontinence, ≥50 % reduction in incontinence episodes per 24 h from baseline to final visit; micturitions, ≤8 micturitions per 24 h at final visit; PPBC, OAB-q symptom bother score, and OAB-q total HRQoL, change from baseline to final visit ≥ minimally important difference (10 points for OAB-q total HRQoL and OAB-q symptom bother score and 1 point for PPBC

^b^95 % two-sided CIs for the differences between mirabegron 50 mg and placebo in proportions of responders are based on normal approximation

^c^Odds ratios, corresponding two-sided 95 % CIs, and *P* values are derived from a logistic regression model including treatment group, sex, study, and baseline value(s)

When responder rates for incontinence episodes were analyzed in combination with responder rates for PPBC, OAB-q symptom bother scale, or OAB-q total HRQoL to identify patients who were simultaneously responders on two (double responder analysis) or three (triple responder analysis) outcomes, mirabegron 50 mg demonstrated statistically significantly greater responder rates compared with placebo in all analyses (*P* < 0.001, Table 3). Likewise, when responder rates for micturition frequency were analyzed in combination with responder rates for one or two PRO outcomes, mirabegron 50 mg demonstrated statistically significantly greater responder rates compared with placebo in all analyses (*P* < 0.001).

#### Correlation analyses

For PPBC and the OAB-q symptom bother scale, a negative change from the baseline value indicates patient improvement; thus, a positive Spearman rank correlation coefficient as shown in Table 4 indicates correlation between the improvement from baseline to final visit for PPBC and that for OAB-q symptom bother scale. Meanwhile, for OAB-q total HRQoL, a positive change from baseline value indicates patient improvement; thus, a negative Spearman rank correlation coefficient as shown in Table 4 indicates correlation between the improvement from baseline to final visit for PPBC and that for OAB-q total HRQoL. Both correlations were statistically significant (*P* < 0.0001). There were also significant correlations between change from baseline to final visit in both objective measures—mean number of incontinence episodes per 24 h and mean number of micturitions per 24 h—and change from baseline to final visit in each of the subjective measures—OAB-q symptom bother scale, OAB-q total HRQoL, and PPBC (*P* < 0.0001, Table 4).

**Table 4:** Spearman rank order correlation coefficients between objective and subjective outcome measures; pooled mirabegron 50 mg and placebo data

	Spearman rank correlation coefficient	*P* value
Correlation between change from baseline to final visit in		
PPBC (FAS) and		
OAB-q symptom bother score	0.62	<0.0001
OAB-q total HRQoL	−0.60	<0.0001
Mean number of incontinence episodes per 24 h (FAS-I) and		
OAB-q symptom bother score	0.40	<0.0001
OAB-q total HRQoL	−0.33	<0.0001
PPBC	0.22	<0.0001
Mean number of micturitions per 24 h (FAS) and		
OAB-q symptom bother score	0.42	<0.0001
OAB-q total HRQoL	−0.37	<0.0001
PPBC	0.31	<0.0001

*FAS* full analysis set, *FAS*-*I* FAS-incontinence, *HRQoL* health-related quality of life, *OAB*-*q* overactive bladder questionnaire, *PPBC* patient perception of bladder condition

## Discussion

These data extend what has been previously published: in a pooled analysis of three 12-week Phase III studies, mirabegron 50 mg not only resulted in statistically significant improvements versus placebo in change from baseline to final visit in the objective outcomes of mean number of incontinence episodes and mean number of micturitions per 24 h, but also resulted in statistically significant improvements versus placebo in change from baseline to final visit in PPBC, the OAB-q symptom bother scale, OAB-q total HRQoL, and OAB-q subscales of coping, concern, and sleeping, but not social interaction. The social interaction subscale has traditionally been the least responsive of the OAB-q subscales to improvement in patients’ wellbeing, so these results are not surprising [35, 36].

The improvements produced by mirabegron 50 mg on PROs were also manifest in the proportion of subjects in whom the change from baseline exceeded the MID defined for both the OAB-q (10 points) and PPBC (1 point) instruments. Indeed, two-thirds of subjects who received mirabegron 50 mg experienced a change in the OAB-q symptom bother scale that exceeded the MID for that scale (10 points). The MID values for OAB-q have been related to changes in degrees of disease symptomatology that are directly relevant to the patient’s experience of the disease, such as resolution of incontinence. Hence, the results demonstrate the meaningfulness of the observed therapeutic benefit experienced by the patient.

There was a significant correlation between change from baseline in PPBC and that in the OAB-q symptom bother scale and OAB-q total HRQoL. Moreover, these correlations were large (0.60 and −0.62) [42] and similar to those between PPBC, OAB-q symptom bother scale, and OAB-q HRQoL reported by Coyne and co-workers [43] in a similar post hoc analysis of data from a 12-week open-label trial of tolterodine extended release. These results indicate directional consistency in the response of these PROs to mirabegron 50 mg.

Incontinence is a major symptom of OAB, occurring in about one-third of patients, and is associated with significant morbidity, reduction in QoL, and serious limitations to activities of daily living [44]. QoL scores in OAB patients with incontinence have been shown to be consistently lower than in OAB patients without incontinence [44]. A statistically significant difference between mirabegron 50 mg and placebo in the percentage of patients who achieved a 50 % or greater reduction in incontinence frequency at final visit has been seen previously [32]. In the post hoc analyses described here, the unique approach was taken to identify the proportion of patients who *simultaneously* achieved a 50 % or greater reduction in incontinence frequency at final visit and the MID in one or two PROs. In all the analyses conducted, mirabegron 50 mg resulted in statistically significantly higher composite responder rates than placebo. Moreover, improvements in both incontinence and micturition frequency were statistically significantly correlated with improvements in each of PPBC, the OAB-q symptom bother scale, and OAB-q total HRQoL. These correlation coefficients were mostly of moderate magnitude (0.30–0.49) [42] and, regardless of which PRO was examined, were similar for incontinence and micturition frequency. A number of studies have demonstrated the favorable effects of several antimuscarinics on a number of different QoL measures [45–49] and a small to moderate, but statistically significant correlation between improvement in number of urgency urinary incontinence episodes and PPBC has been reported in a 12-week trial of tolterodine extended release [50]. However, no studies of antimuscarinics have evaluated composite responder rates as done here.

In conclusion, it appears that the improvements in objective outcome measures seen with mirabegron 50 mg are mirrored by statistically significant improvements in the clinically relevant PRO measurements of OAB-q total HRQoL, PPBC, and OAB-q symptom bother scale. Moreover, there is directional consistency in the effect of mirabegron 50 mg across the disparate domains measured by various objective outcomes and PROs. Thus, improvement in objective outcomes translates into a meaningful clinical benefit; the OAB-q and PPBC appear to provide a clinically relevant perspective on OAB management, and the objective outcomes of incontinence and micturition frequency appear to be adequate proxies of patient experience. The results bolster the view that mirabegron may be a good treatment option for OAB patients as improvements in HRQoL measures are likely to be reflected in high patient satisfaction. Nonetheless, studies that specifically evaluate patient satisfaction are necessary to confirm this suggestion. In addition, studies examining patient adherence with mirabegron are required; patient adherence with antimuscarinics, the current mainstay of treatment for OAB, is poor, largely due to intolerable side effects, and it will be interesting to see whether mirabegon’s favorable effects on QoL measures translate into good adherence levels.
